# Exploring the digital divide: results of a survey informing mobile application development

**DOI:** 10.3389/fdgth.2024.1382507

**Published:** 2024-05-10

**Authors:** Maira Corinne Claudio, Zachary Rehany, Katerina Stachtari, Elena Guadagno, Esli Osmanlliu, Dan Poenaru

**Affiliations:** ^1^ Faculty of Medicine and Health Sciences, McGill University, Montreal, QC, Canada; ^2^ Harvey E. Beardmore Division of Pediatric Surgery, The Montreal Children’s Hospital, McGill University Health Center, Montreal, QC, Canada; ^3^ Division of Pediatric Emergency Medicine, Department of Pediatrics, The Montreal Children’s Hospital, McGill University Health Centre, Montreal, QC, Canada

**Keywords:** digital divide, pediatric, mobile applications, telemedicine, health equity

## Abstract

**Introduction:**

Mobile health apps risk widening health disparities if they overlook digital inclusion. The digital divide, encompassing access, familiarity, and readiness, poses a significant barrier to medical interventions. Existing literature lacks exploration of the digital divide's contributing factors. Hence, data are needed to comprehend the challenges in developing inclusive health apps.

**Methods:**

We created a survey to gauge internet and smartphone access, smartphone familiarity, and readiness for using mobile health apps among caregivers of pediatric patients in tertiary care. Open-ended questions solicited feedback and suggestions on mobile health applications. Responses were categorized by similarity and compared. Developed with patient partners, the survey underwent cognitive testing and piloting for accuracy.

**Results:**

Data from 209 respondents showed that 23% were affected by the digital divide, mainly due to unfamiliarity with digital skills. Among 49 short text responses about health app concerns, 31 mentioned security and confidentiality, with 7 mentioning the impersonal nature of such apps. Desired features included messaging healthcare providers, scheduling, task reminders, and simplicity.

**Conclusions:**

This study underscores a digital divide among caregivers of pediatric patients, with nearly a quarter affected primarily due to a lack of digital comfort. Respondents emphasized user-friendliness and online security for health apps. Future apps should prioritize digital inclusion by addressing the significant barriers and carefully considering patient and family concerns.

## Introduction

Mobile health applications (“apps”) are gaining popularity for patient services due to their practicality in enabling remote patient contact and reducing the need for in-person follow-up, thereby alleviating medical or operative complications and improving overall care outcomes (1, 2). By prioritizing inclusivity, mobile health innovations can effectively address the diverse needs of users and promote equitable healthcare outcomes (3, 4).

The *digital divide* refers to the gap that exists between individuals who have adequate digital access and those who do not (5). The digital divide also extends to include those who lack the necessary proficiency or motivation to use digital tools (6, 7). We have categorized the factors causing the digital divide into three main categories based on recurring themes in the literature, which are digital access, familiarity, and readiness. We then explain the relevance these categories have concerning mobile health apps.

### Access

Access to mobile health apps is a critical aspect of addressing disparities in healthcare delivery. Limited access to smartphones and reliable internet can impede individuals from using health apps, particularly those from marginalized communities or underserved regions. Although trends show that the level of smartphone and internet ownership is rapidly increasing, there is still a portion of the population who still face barriers to access (8, 9).

### Familiarity

Health disparities can also increase if individuals lack the proficiency to use these tools. The usability of an app is therefore very important when implementing mobile health apps. Studies find that health apps with poor rated usability were much less commonly adhered to and therefore would have little impact on the level of care (2, 10). In many cases, health apps have caused stress or frustration among patients because of design or navigational issues thereby causing reluctance to use them (10, 11). Another study revealed that around 1 in 3 adults are not aware of in-app customizations leading many to feel that the apps did not fit their needs (12).

### Readiness

A person's willingness to use a mobile health app also plays a critical role in the digital divide. Having a poor understanding of the value of mobile interventions lead users to have a lower motivation in deciding to use them (7). A study analyzing the factors impacting the use of mobile health apps found that usability alone isn't sufficient to motivate patients in using them (12). There are numerous hesitations people may have that prevent them from engaging in health apps such as lacking confidence in the benefits, finding them tedious, and having data privacy or confidentiality concerns (7, 13, 14). Meanwhile, greater patient engagement has been found to be improved by raising awareness of health apps through more clinical endorsements and physician recommendations (12, 15, 16).

The objective of this study is to examine the digital divide by measuring the extent of the major contributing factors. This was done by administering a survey at our site with multiple-choice questions for access, familiarity and readiness categories. To further assess the quality of patients' readiness, we introduced the concept of a health app and asked open-ended questions about their concerns or what they would find valuable in such an app. Consequently, the survey results will yield an overall picture of the digital divide's characteristic nature and barriers, which will inform the development and implementation of health apps in ensuring inclusivity. We have not found a study in the literature that has integrated all three factors in order to examine the overall impact of the divide. By doing so, similar institutions can gauge the level of effort needed to address each barrier and enhance these tools accordingly for broader accessibility.

## Methods

A cross-sectional survey was conducted at the Montreal Children's Hospital (MCH), an urban tertiary pediatric hospital that serves a multicultural population, encompassing patients from multiple cultural backgrounds and ethnicities typically cared for at our institution. In the context of this study, we aimed to identify constraints that may limit patients' ability to use digital health interventions. Eligible participants for the study were caregivers (including parents and guardians) of children under 18 years old who had visited the MCH Pediatric Surgery outpatient clinic or Emergency Department. Participants were required to be able to complete the survey in either English or French. The study recruited a convenience sample of caregivers at the locations between January and May 2022. The surveys were accessible either through stands placed in these locations or by being distributed by department staff members. Additionally, participants had the choice to complete either a paper survey or an online version by scanning a QR code. Respectively, the decision to collect survey responses via both QR code and paper format stemmed from a recognition of the varying digital literacy levels among respondents. Specifically, it is important to acknowledge that individuals proficient in digital technologies may be less likely to utilize the paper version of the survey. As a result, presenting responses from both groups becomes informative and enhances the comprehensiveness of the study. Ethical approval for the study was obtained from the McGill University Health Centre's Research Ethics Board (REB 2022-8127).

The survey instrument was developed by adapting questions from two validated instruments: Canadian Internet Use Survey (CIUS) and eHealth Literacy Questionnaire (eHLQ) (17, 18).

The Canadian Internet Use Survey (CIUS) is intended to examine the Canadian population over the age of 15 to better understand how digital technology affects people's lives (17). It encompasses internet-connected smart devices, digital skills, and security elements such as privacy and trust, among others. Furthermore, it assesses barriers to internet access and the usage of digital technology. The survey is meant to inform evidence-based policymaking on digital technology among the Canadian population. The eHealth Literacy Questionnaire (eHLQ) is a developed validated tool that is based on the eHLF framework (18). The eHLQ takes into consideration health literacy and the need to assess that the rapid advancement of digital health literacy is meeting user needs and unique experiences. As a result, both tools were chosen based on their reliability and alignment with our priorities to study the digital divide at the MCH, which are to reduce access obstacles and better understand the use of digital and internet access, alongside concerns among users.

To ensure the study's quality, feedback from team interactions was incorporated, following the experience-based co-design method, which is an approach aimed at improving healthcare services by involving both the stakeholders (patients/caregivers) and physicians in the design process (19). In our study, this included a group of patient research partners with whom we discussed each step of the project proposal, survey development and the analysis of the collected data. In the cognitive testing stage, five patient partners reviewed and assessed the survey for question interpretation ambiguities, leading to modifications for improved comprehensibility. A pilot test of the survey was also conducted with ten individuals, and the final draft was discussed in sessions with small groups of patient partners. The patient partners were members of the lab's Patient Advisory Committee, composed of parents/caretakers of children treated in the Department of Surgery.

The structured questionnaire comprised three domains: digital access, familiarity, and readiness. The digital access domain assessed participants' level of home or mobile internet, as well as smartphone access. The digital familiarity domain measured participants' comfort or skill level in using a smartphone, including their knowledge of downloading smartphone apps and familiarity with common smartphone features. The readiness domain sought patient perspectives/attitudes on mobile healthcare apps. A respondent was deemed affected by the digital divide if they showed vulnerability in at least one domain. To further assess the respondents' attitudes toward mobile health apps, a concept test of a pediatric health app was presented, including questions about features they would find valuable in such an app and any concerns they may have (viewable in Appendix 1). Cohort characteristics were measured, including age, education, employment status and preferred language, but were deliberately kept separate from the survey data to prioritize respondent privacy.

**Figure 1:**
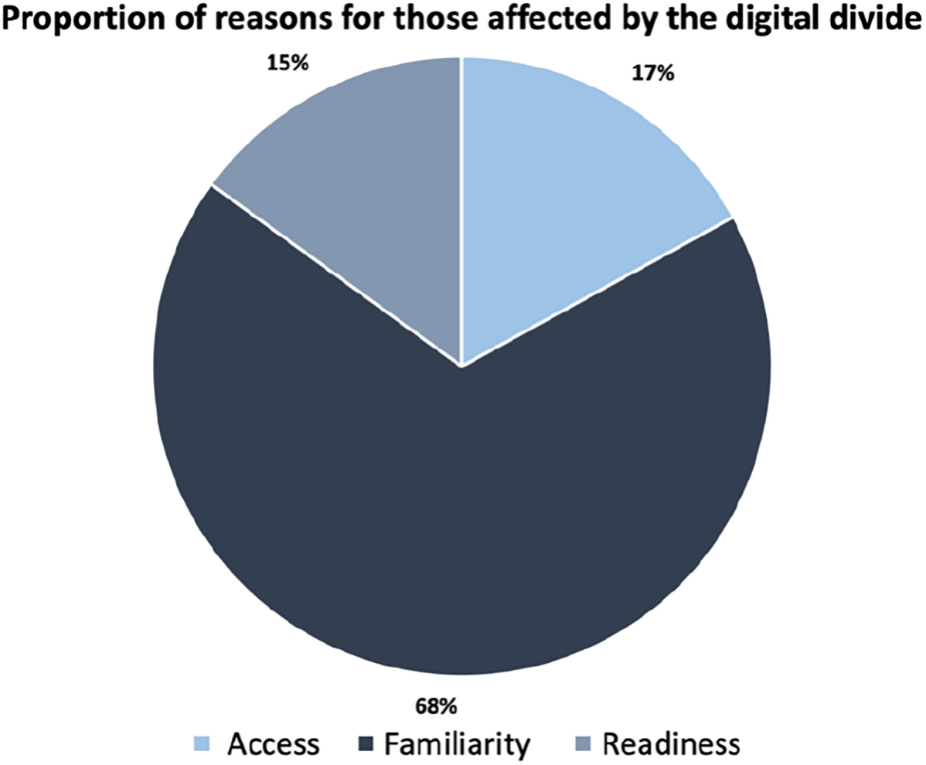
The classification of different reasons for being affected by the digital divide. Individuals affected by a combination of factors were accounted for once and overlaps were resolved.

In order to quantify the significance of each of the 3 domains, we selected specific questions within each category that provided a clear indication of impact, while excluding those with potential for ambiguous interpretation. Lack of access was determined by whether participants lacked adequate home internet or smartphone ownership (Q1 & 3). Lack of digital familiarity by considering participants who expressed discomfort using smartphones or were unsure how to download smartphone apps (Q6 & 7). Lastly, a lack of readiness by identifying participants who expressed unwillingness to use mobile apps for their health (Q9). When classifying the total affected individuals into the three domains (shown in Figure 1), an individual was counted once per domain to prevent overlapping in the results. This approach ensured that the final count accurately represented the number of individuals affected by the digital divide, without inflating the numbers due to overlapping categories or double counting.

Descriptive analysis was performed on the multiple-choice results, while analyses of short-text answers involved grouping similar responses into themes and comparing the number of responses within each theme. This analysis was done manually, without utilizing automated coding techniques. This approach ensured a meticulous examination of the data. An example of classing two responses into the same theme would be: “the privacy of information given and protection from hackers” and “personal information being shared & security concerns” were both classified into the theme of security & confidentiality.

## Results

There were 209 completed surveys (104 on paper, 105 online). Out of the 185 participants who provided their primary language information, 103 (56%) identified as anglophone, while 76 (41%) identified as francophone (Table 1). We did not collect information regarding the sex of respondents. Additionally, respondents were only asked to indicate their age from the provided age ranges, as seen in Table 1. Further demographic data can also be found in Table 1.

**Table 1:** Summary of respondents’ demographics.

	Digital	Paper	Count
Age			*n* = 186
14–17	1	0	1 (1%)
18–24	16	0	16 (9%)
25–34	17	20	37 (20%)
35–44	35	41	76 (41%)
45–54	16	33	49 (26%)
55–64	0	3	3 (2%)
65–74	1	1	2 (1%)
75 or over	0	2	2 (1%)
Education completed			*n* = 184
High school certificate or equivalent	22	35	57 (31%)
Undergraduate	35	41	76 (41%)
Postgraduate	16	14	30 (16%)
No certificate, diploma, degree	8	1	9 (5%)
I prefer not to answer	2	3	5 (3%)
Other	3	4	7 (4%)
Employment status			*n* = 186
Full-time (over 30 h/week)	50	68	118 (63%)
Part-time (under 30 h/week)	10	13	23 (12%)
Unemployed	3	9	12 (6%)
Retired	1	2	3 (2%)
Student	17	0	17 (9%)
I prefer not to answer	3	2	5 (3%)
Other	2	6	8 (4%)
Preferred language of communication			*n* = 185
English	42	61	103 (56%)
French	41	35	76 (41%)
Other	3	3	6 (3%)

Non-respondents for each question were excluded from the data.

Five respondents (2%) reported not having internet access at home. Among those with internet access, 7 respondents (3%) rated their connectivity as “bad” or “very bad”. Eight respondents (4%) reported not having a mobile internet plan while 4 (2%) were unaware whether they did or not. Five participants (2%) did not own a smartphone, all of whom were above the age of 45. The primary reasons for not having internet access or a smartphone were expenses and/or lack of interest. Of the five individuals without a smartphone, three had a household member who did own one. Among smartphone owners, 29 respondents (13%) reported discomfort in using their smartphones, and 9 respondents (4%) were unaware of how to download an app. Additionally, 15 respondents (7%) expressed a lack of knowledge regarding any of the most common smartphone apps or features.

When participants were presented with the concept of a healthcare app, 183 participants (96%) affirmed that they would use the app if it was available and valuable to them and their families. The majority of respondents expressed willingness to communicate with their healthcare providers (94%) and to share their medical information through an app (92%). A detailed summary of all the findings is presented in Table 2.

**Table 2:** Summary of respondent answers.

Theme	Survey question (*n* = total responses)	Response option	Digital	Paper	Count
Digital access	Q1 Internet access at home (*n* = 209)	Yes	105	99	204 (98%)
		No	0	5	5 (2%)
	Q2 Internet connectivity rating (*n* = 203)	Very Bad	0	4	4 (2%)
		Bad	2	1	3 (1%)
		Good	47	35	82 (40%)
		Very Good	54	60	114 (56%)
	Q2.1 *Reasons for not owning internet connectivity (*n* = 5)	Too expensive	0	4	4 (80%)
		Too difficult to use	0	0	0 (0%)
		No need or interest	0	1	1 (20%)
		Other	0	0	0 (0%)
	Q3 Access to a smartphone (*n* = 209)	Yes	105	99	204 (98%)
		No	0	5	5 (2%)
	Q4.0 Mobile internet on smartphone plan (*n* = 203)	Yes	101	90	191 (94%)
		No	2	6	8 (4%)
		Unsure	2	2	4 (2%)
	Q4.1 *Reasons for not owning a smartphone (*n* = 5)	To expensive	0	2	2 (40%)
		Safety and privacy concerns	0	1	1 (20%)
		Unaware how to use a smartphone when needed	0	1	1 (20%)
		When needed			
		Access to another smartphone	0	1	1 (20%)
		When needed			
		No need or interest	0	4	4 (80%)
		Other	0	0	0 (0%)
	Q5 Members in household familiar with using smartphones (*n* = 209)	Yes	104	92	196 (94%)
		No	1	12	13 (6%)
Digital familiarity	Q6 Level of comfort using a smartphone (*n* = 204)	Very uncomfortable	10	13	23 (11%)
		Uncomfortable	2	3	6 (3%)
		Comfortable	24	35	59 (29%)
		Very comfortable	67	49	116 (57%)
	Q7 Awareness on how to download an app (*n* = 201)	Yes	104	88	192 (96%)
		No	0	9	9 (4%)
	Q8 *Smartphone uses in the last month (*n* = 203)	Daily news	77	69	146 (72%)
		Social media	91	85	176 (87%)
		Video calls	90	78	168 (83%)
		Directions	86	82	168 (83%)
		Email	82	79	161 (79%)
		Banking	71	67	138 (68%)
		Internet Searches	89	87	176 (87%)
		Movies	67	52	119 (59%)
		None of the above	14	1	15 (7%)
Patient readiness	Q9 If this app was available to me, I would likely use it (*n* = 191)	Strongly disagree	2	2	4 (2%)
		Disagree	1	3	4 (2%)
		Agree	24	36	60 (31%)
		Strongly agree	63	60	123 (64%)
	Q10 This app would be important to my family and I (*n* = 191)	Strongly disagree	2	2	4 (2%)
		Disagree	3	2	5 (4%)
		Agree	36	46	82 (43%)
		Strongly agree	49	51	100 (62%)
	Q11 I would be willing to communicate with my doctor online through a mobile health app like this one (*n* = 191)	Strongly disagree	2	2	4 (2%)
		Disagree	3	4	7 (4%)
		Agree	28	34	62 (32%)
		Strongly agree	57	61	118 (62%)
	Q12 I would be comfortable with sharing information with my doctor on this mobile health app (*n* = 191)	Strongly disagree	2	4	6 (3%)
		Disagree	7	3	10 (5%)
		Agree	27	46	73 (38%)
		Strongly agree	53	49	102 (53%)

Non-respondents for each question were excluded from the data. For purposes of this table, questions were simplified.

* Multiple responses were permitted

Overall, 23% of respondents were affected by the digital divide to some extent. This was based on 10 people being affected by access, 38 affected by familiarity, and 8 affected by readiness, from the 5 selected questions. There were a total of 10 individuals who belonged to multiple groups, and therefore were only counted once to avoid overlapping results. Consequently, as seen on Figure 1, the classification of the total is as follows: 17% due to a lack of access, 68% due to a lack of familiarity, and 15% based on readiness.

With regards to open-ended questions, the most frequently mentioned concerns related to app usage were security and confidentiality, as identified in 31 out of the 49 short-text responses. For instance, a comment regarding confidentiality is the “*Concern over cybersecurity and the app's ability to safeguard private and personal information*”. The next most popular concern was the potential impersonal nature of the app, which was included in 7 of the short answer responses. One such comment made regarding this aspect was “*I would still always like to have human contact. A machine cannot replace a doctor.*” Finally, common participant suggestions for app features included efficient communication with physicians, appointment scheduling, task reminders, access to medical results and, most commonly, user-friendliness and app simplicity. Respectively, some comments made are as follows: suggestions for communication were made, with one participant suggesting: “*Being able to send my Doctor messages about changes or updates. Being able to ask questions and have the doctor's responses”. Additionally*, concerns regarding digital literacy were raised, with another participant mentioning, “*Providing direct access is crucial, especially considering that these apps can become complex for older adults and non-tech savy users*.” Moreover, participants stressed the significance of user comfort and ease, as indicated by a comment suggesting that “*The app's interface should prioritize clarity and brevity to enhance user experience*.” Finally, for features, participants recommended a range of suggestions, including “*Offering appointment planning and reminders, providing preparation information, offering condition-specific insights, and including prescription details and instructions on medication usage.*”

## Discussion

Digital technologies, such as mobile health apps, may exacerbate health disparities if they overlook digital inclusion in their design. Our study evaluated the digital divide in terms of digital access, familiarity and readiness at a tertiary care institution, particularly regarding mobile health apps, which has not been previously addressed in the literature. We discovered that at least 23% of the institution's patient population faces the digital divide. A lack of digital familiarity was the principle barrier measured in this percentage, while the vast majority of respondents had access and had ready attitudes to use mobile health apps. Having this understanding of the extent of each of these barriers will help guide future app developers on where to focus their efforts to improve inclusivity.

In terms of digital access, most families at our pediatric institution had a greater proportion of internet and smartphone access compared to the overall North American population. Specifically, our site had 98% access to both internet and smartphones, while the North American average ranged from 85% to 95% in 2021 (8, 20). A study in the U.S. revealed that 16% of Americans aren't digitally literate, while 19% of respondents at our institution reported discomfort in using their smartphones or being unaware of how to download an app (21). Statistics Canada data showed that 97% of individuals between the ages of 25–44 have access to a smartphone, whereas 87% of those aged 45–65 do not (22). In our study, those who did not own a smartphone in our population were predominantly above the age of 45, similarly indicating that older age is a likely factor for not having access to a smartphone. Additionally, our study found that over half of the individuals without a smartphone had a household member who possessed one, suggesting a potential compensatory approach for the lack of personal access to a smartphone. The results above reveal that our patient population had a high level of digital access, especially compared to the general population. We therefore consider digital access as a minor barrier to health app inclusion.

In our findings, the most remarkable factor, potentially preventing those to benefit from health apps, is the lack of smartphone familiarity. Apps ought to be easy to use, as we find in the literature that health apps with low usability ratings are linked to poor adherence, limiting their impact on patient care, and design issues often lead to patient frustration, reducing their benefit to users (10, 11). Among the short text responses, the importance of ease of use in health apps was strongly emphasized by our participants, suggesting that simplicity should take precedence over multi-functionality in app development. This also suggests the need for technical onboarding and app tutorials for first time users as well as piloting the app to assess its perceived ease of use.

In order to address the lack of digital familiarity among users, app usability should be properly assessed. In a systematic review of 33 studies evaluating the usability of mHealth apps, it has been found that current app reviews for usability are substandard (2). Many surveys focus on assessing satisfaction rather than usability. Methodologies of assessing the usability were considered poor, using self-made questionnaires, and having conflict of interests. An example of a reliable validated tool to evaluate health app usability is the mHealth App Usability Questionnaire (MAUQ) (23).

In order to characterize users' level of comfort, another study in the literature used an interesting method where participants were inquired about their expectations of health apps (24). This specifically included asking about desired features, intentions to use, perceived aesthetics, ease of use, and usefulness of health apps, all of which can be dependent on a person's digital skills or comfort. They were then presented with apps that varied in similarity to their expectations, ranging from low to high. The study found that health apps that matched user expectations increased their willingness to adopt them and their perception of value during actual use. This underscores the importance for app developers to evaluate the expectations of their target audiences in order to meet their needs effectively in the design considerations.

Despite facing various barriers related to digital access and familiarity, nearly all respondents expressed their willingness to use a mobile health app if it were available, as evident from their responses in the readiness domain. This observation highlights that individuals affected by the digital divide display a strong inclination toward digital health participation. However, this finding is assumed to be on the premise of an ideal mobile health app perceived by the responders, with the app's usability and functions meeting their expectations. It is therefore important to understand the concerns and suggestions patients made regarding such apps.

In our study, we collected interests and concerns related to health apps from respondents at our tertiary care institution in order to tailor future apps to their needs and increase overall app use. Comparing our results to previous studies, we found similar perspectives/attitudes among participants regarding the engagement with mobile health apps. In our study, concerns related to security, privacy, and ease of use were consistently mentioned, reflecting the importance of addressing these aspects in app development. Studies have shown that patients often lack trust in health apps and other online portals (12). Digital literacy also plays a crucial role in understanding security policy complexities (22, 25). A systematic review of factors contributing to limited patient use of health apps suggests that a lack of trust and clinical support for mobile apps pose significant barriers to patient engagement (6). Meanwhile, physician recommendations and clinical endorsements of health apps have been shown to increase patient interest and confidence in using them (12, 15, 16). App developers should be totally aware of this potential barrier for users. Ultimately, strengthening app security, addressing user safety during recruitment, and seeking healthcare provider endorsements can help overcome confidence barriers and narrow the digital divide.

A study measuring factors impacting the use of health apps found that patients reported that their likelihood of using such apps is also influenced by their perceived benefits or risks and not solely by usability (12). In our study, we gathered patient recommendations for what they believe are important app features. These included having a communication platform with their physicians, the option for appointment booking, task reminders such as for their treatments, and access to their medical results. We found that patients most commonly requested the apps be user-friendly and simple. In order to maximize the use and perceived importance of a health app, it is important to consider the value it has for patients, otherwise, it can be considered a burden to use.

In the context of pediatrics, mobile health apps have successfully shown to help preoperative and postoperative stress in pediatric patients (26). Meanwhile, these apps often prioritize caregivers over pediatric patients (27). This approach aligns with efforts to promote digital inclusion, recognizing that a child's access to health apps is heavily influenced by socio-economic factors, including internet access and device availability (26). Consequently, minors typically rely on caregivers' devices and internet access for app usage.

In our study, respondents emphasized their concern regarding the impersonal nature that health apps may introduce. This concern is particularly significant in pediatrics, where the doctor-patient relationship plays a crucial role in improving pediatric health outcomes (28). Health app developers ought to be mindful of this aspect, ensuring that their apps facilitate personal connections between physicians and patients, rather than replacing or diminishing the importance of this relationship.

Although our study did not inspect epidemiological correlations, it is worth noting that a recent systematic review of digital disparities during the COVID-19 pandemic highlighted lower digital skill sets among minority groups and the elderly (29). Disparities in digital access were also found to be influenced by factors such as race, minority status, and income group (30).

There are several limitations to our study. Firstly, being a survey-based study, it had a limited sample size and was susceptible to selection bias, as those who chose to fill out the survey may not be representative of the entire study population, especially outside urban tertiary care facilities. Furthermore, the survey was only offered in English and French, which may have excluded individuals who are not proficient in either of these languages. Further research is needed to identify the digital inclusion needs of families who face language barriers, as they may already experience disparities in traditional healthcare access.

It is worth acknowledging that the actual number of people affected by the digital divide may be higher than our estimation. This discrepancy can arise because we selected questions with unambiguous responses when estimating the number of people affected. For example, our calculations include only individuals with no access to the internet, while in reality, those with very poor connectivity may also be impacted by the digital divide but were not accounted for in our estimation.

In conclusion, this study evaluated the digital divide based on access to digital technologies, familiarity with their use, and willingness to use them for health purposes. As a result, we found that nearly a quarter of patrons at our tertiary care pediatric institution face at least one barrier to using a mobile app for health. Additionally, the study identified the values and preferences of parents or caregivers of pediatric patients when deciding to use a health app, including the desire for a simple and secure app with specific features. Being informed with this information, health app developers can better understand how to enhance the usability and relevance of their apps, ultimately bridging the digital divide and promoting equitable healthcare access.

## Data Availability

The original contributions presented in the study are included in the article/Supplementary Material, further inquiries can be directed to the corresponding author.

## Appendix 1. Administered Questionnaire

### Internet access and smartphone use of patient caretakers

The purpose of this survey is to understand your readiness and access to digital devices, such as with the internet and cell phones. Your participation will help create a tool that improves the communication between families and pediatric care teams. We value your involvement as a patient or caregiver at the Montreal Children's Hospital.

The questionnaire is anonymous and voluntary: we will not know which responses are yours and you may stop responding at any time. *By completing this questionnaire, you acknowledge that you have read, understood, and agreed with the above information. Your choice to participate or not will not affect the care that you or your child(ren) receive at this hospital*.

If you have any questions about this research project, please contact the lead clinician: Dr. Dan Poenaru—dan.poenaru@mcgill.ca. The survey has 19 questions and should take 10 min to complete.

You may choose to complete this questionnaire online. To do so, open the Camera app on your phone and point the camera towards this image:

Useful terms:

A **Cell or Mobile Phone** is a handheld device that can make and receive calls. These phones have a number pad or keyboard (for example: flip phone or Nokia).

A **Smartphone** is a type of cell phone that has a touch screen, is able to use the internet for looking things up online and that you can watch videos or play games (for example: iPhone, Android, Google Phone).

A **Mobile Health App** is a health related service offered on a smartphone. It can be used for communicating with a doctor, recording diets, measuring heart rate or blood sugar, fitness/exercising and medication reminders.

**A. Digital Access—We would like to know about your access to the internet and to a smartphone.**

1. Do you have internet access at home?

**Table:**

▢ Yes	▢ No
2. *If Yes:* How would you rate your internet connectivity (speed, stability, etc.)? ▢ Very bad ▢ Bad ▢ Good ▢ Very good	2. *If No:* What are the reasons for not having access to the internet? *Check all that apply:* ▢ Too expensive ▢ Too difficult to use ▢ No need or interest ▢ Other: _____________

3. Do you have a smartphone (example: iPhone, Android, Samsung Galaxy, Google Phone, etc.)?

**Table:**

▢ Yes	▢ No
*4. If Yes:* Do you have Mobile Internet (cellular data) on your smartphone plan? ▢ Yes ▢ No ▢ I am unsure	*4. If No:* What are the reasons for not owning a smartphone? *Check all that apply:* ▢ Too expensive ▢ I’m concerned about data safety and privacy ▢ I don’t know how to use a smartphone ▢ I use someone else's smartphone if needed ▢ No need or interest ▢ Other: _________________

5. Do you have other members in your household who are familiar with using smartphones?

▢ Yes ▢ No

**B. Digital Skills & Familiarity—We would like to know how well you can use a smartphone.**

*If you do not own a smartphone, continue to Section C*.

6. How comfortable do you feel when you use a smartphone? *Mark your answer on the scale below:*

▢ 1 - Very uncomfortable ▢ 2 - Uncomfortable ▢ 3 - Comfortable ▢ 4 - Very comfortable

7. Do you know how to download an App on a smartphone?

▢ Yes ▢ No

8. In the last month, what have you used your smartphone for? *Check off all that apply:*

▢ Daily news ▢ Social media ▢ Video calls ▢ Directions ▢ Email

▢ Banking ▢ Internet searches ▢ Movies ▢ None of the above

**C. Patient perspectives—We would like to know your opinions on the following:**

The Department of Pediatrics at Montreal Children's Hospital is developing a smartphone App to improve communication around the experience of medical or surgical care. We hope that it will decrease anxiety and increase the comfort of patients and their caregivers.

The app will:

1) Give parents/caregivers access to communicate directly with their medical or surgical team
2) Help with planning and providing reminders for appointments
3) Provide educational information on relevant medical/surgical conditions

*Please rate your level of agreement with the sentences below, using a scale from 1 to 4:*

9. If this app was available to me, I would likely use it.

▢ 1 - Strongly disagree ▢ 2 - Disagree ▢ 3 - Agree ▢ 4 - Strongly agree

10. This app would be important to me or my family.

▢ 1 - Strongly disagree ▢ 2 - Disagree ▢ 3 - Agree ▢ 4 - Strongly agree

11. I would be willing to communicate with my doctor online through a mobile health app like this one.

▢ 1 - Strongly disagree ▢ 2 - Disagree ▢ 3 - Agree ▢ 4 - Strongly agree

12. I would be comfortable sharing medical information with my doctor on this mobile health app.

▢ 1 - Strongly disagree ▢ 2 - Disagree ▢ 3 - Agree ▢ 4 - Strongly agree

13. Are there any concerns you have about this app?

14. What features would you like to see in this app?

**D. Personal Information—We would like to know more about you.**

*We'd like to remind you that this survey is anonymous. We will not know which responses are yours*.

15. Which age group do you belong to?

▢ 14–17 ▢ 18–24 ▢ 25–34 ▢ 35–44 ▢ 45–54 ▢ 55–64 ▢ 65–74 ▢ 75 or over

16. Which of the following best describes your employment status at the present time? *If you are on sabbatical, maternity leave, sick leave or work-related accident leave, indicate whether you usually work full-time or part-time*.

▢ I work full-time (over 30 h/week) ▢ I work part-time (under 30 h/week)

▢ I am unemployed ▢ I am retired

▢ I am a student ▢ I prefer not to answer

▢ Other: ________________

17. What is the highest level of education you have completed?

▢ High School Certificate or Equivalent

▢ Undergraduate Degree (BA, BSc, etc.)

▢ Postgraduate Degree (Masters, PhD, MD, etc.)

▢ No certificate, diploma, or degree

▢ I prefer not to answer

▢ Other: _____________

18. What is your preferred language of communication?

▢ English ▢ French ▢ Other: __________

19. What are the first three characters of your postal code? _____________

*Thank you for taking the time to complete this survey! We truly value the information you have provided—your responses will help improve surgeons' communication with children and their close ones*.

Would you like to be part of the team by sharing your expertise as a patient or caregiver? If so, we will invite you to meet with our team and help us design a user-friendly app for patients using the results of this survey.

If so, please enter your email address or phone number for us to contact you: ______________________________.

## References

[1] Timmers T Janssen L Kool RB Kremer JA. Educating patients by providing timely information using smartphone and tablet apps: systematic review. J Med Internet Res. (2020) 22(4):e17342. 10.2196/17342 32281936 PMC7186866

[2] Patel B Thind A. Usability of mobile health apps for postoperative care: systematic review. JMIR Perioper Med. (2020) 3(2):e19099. 10.2196/19099 33393925 PMC7709840

[3] Veinot TC Mitchell H Ancker JS. Good intentions are not enough: how informatics interventions can worsen inequality. J Am Med Inform Assoc. (2018) 25(8):1080–8. 10.1093/jamia/ocy052 29788380 PMC7646885

[4] Mackert M Mabry-Flynn A Champlin S Donovan EE Pounders K. Health literacy and health information technology adoption: the potential for a new digital divide. J Med Internet Res. (2016) 18(10):e264. 10.2196/jmir.6349 27702738 PMC5069402

[5] Dictionary.com. Digital divide definition & meaning. (2022). Available online at: https://www.dictionary.com/browse/digital-divide (Accessed November 8, 2022).

[6] Benton Institute for Broadband & Society. The Digital Skill Divide. (2023). Available online at: https://www.benton.org/blog/digital-skill-divide#:~:text=The%20digital%20skill%20divide%20is,and%20those%20who%20do%20not (Accessed March 22, 2024).

[7] O'Connor S Hanlon P O'Donnell CA Garcia S Glanville J Mair FS. Understanding factors affecting patient and public engagement and recruitment to digital health interventions: a systematic review of qualitative studies. BMC Med Inform Decis Mak. (2016) 16(1):120. 10.1186/s12911-016-0359-3 27630020 PMC5024516

[8] Greenwood S. “Mobile technology and home broadband 2021,” (2021). Available online at: https://www.pewresearch.org/internet/2021/06/03/mobile-technology-and-home-broadband-2021/ (Accessed June 22, 2021).

[9] Pew Research Center. Mobile fact sheet. Washington, DC (2017). Available online at: http://www.pewinternet.org/fact-sheet/mobile/ (Accessed January 15, 2017).

[10] Sarkar U Gourley GI Lyles CR Tieu L Clarity C Newmark L Usability of commercially available mobile applications for diverse patients. J Gen Intern Med. (2016) 31(12):1417–26. 10.1007/s11606-016-3771-6 27418347 PMC5130945

[11] Meirte J Hellemans N Anthonissen M Denteneer L Maertens K Moortgat P Benefits and disadvantages of electronic patient-reported outcome measures: systematic review. JMIR Perioper Med. (2020) 3(1):e15588. 10.2196/15588 33393920 PMC7709853

[12] Scott AR Alore EA Naik AD Berger DH Suliburk JW. Mixed-Methods analysis of factors impacting use of a postoperative mHealth app. JMIR Mhealth Uhealth. (2017) 5(2):e11. 10.2196/mhealth.6728 28179215 PMC5322201

[13] Semple JL Armstrong KA. Mobile applications for postoperative monitoring after discharge. CMAJ. (2017) 189(1):E22–4. 10.1503/cmaj.160195 27920015 PMC5224949

[14] Nielsen AS Kidholm K Kayser L. Patients’ reasons for non-use of digital patient-reported outcome concepts: a scoping review. Health Inform J. (2020) 26(4):1460458220942649. 10.1177/1460458220942649 32731773

[15] Szinay D Jones A Chadborn T Brown J Naughton F. Influences on the uptake of and engagement with health and well-being smartphone apps: systematic review. J Med Internet Res. (2020) 22(5):e17572. 10.2196/17572 32348255 PMC7293059

[16] Peacock S Reddy A Leveille SG Walker J Payne TH Oster NV Patient portals and personal health information online: perception, access, and use by US adults. J Am Med Inform Assoc. (2017) 24(e1):e173–7. 10.1093/jamia/ocw095 27413120 PMC7651932

[17] Statistics Canada. Canadian internet use survey (2020). Available online at: https://www23.statcan.gc.ca/imdb/p3Instr.pl?Function=assembleInstr&lang=en&Item_Id=1289522#qb1290345 (Accessed July 15, 2021).

[18] Kayser L Karnoe A Furstrand D Batterham R Christensen K Elsworth G “A multidimensional tool based on the eHealth literacy framework: development and initial validity testing of the eHealth literacy questionnaire” (eHLQ). J Med Internet Res. (2018) 20(2):e36. 10.2196/jmir.8371 29434011 PMC5826975

[19] EBCD: Experience-based co-design toolkit. (n.d). The point of care foundation. Available online at: https://www.pointofcarefoundation.org.uk/resource/experience-based-co-design-ebcd-toolkit/ (Accessed March 22, 2024).

[20] Government of Canada, Statistics Canada. “Access to the internet in Canada, 2020,” (2021). Available online at: https://www150.statcan.gc.ca/n1/daily-quotidien/210531/dq210531d-eng.htm (Accessed June 29, 2021).

[21] Pawlowski E Hudson L. “A description of US adults who are not digitally literate,” NCES stats in brief, no. 2018-161, Washington: National Center for Education Statistics (2018). Available online at: https://nces.ed.gov/pubsearch/pubsinfo.asp?pubid=2018161 (Accessed June 15, 2021).

[22] Statistics Canada. Table 22-10-0113-01, use of internet services and technologies by age group and household income quartile (2019). (Accessed July 15, 2021). 10.25318/2210011301-eng

[23] Zhou L Bao J Setiawan IMA Saptono A Parmanto B. The mHealth app usability questionnaire (MAUQ): development and validation study. JMIR Mhealth Uhealth. (2019) 7(4):e11500. 10.2196/11500 30973342 PMC6482399

[24] Lazard AJ Babwah Brennen JS Belina SP. App designs and interactive features to increase mHealth adoption: user expectation survey and experiment. JMIR Mhealth Uhealth. (2021) 9(11):e29815. 10.2196/29815 34734829 PMC8603164

[25] Pawlowski E Hudson L. “A description of US adults who are not digitally literate.” NCES stats in brief, no. 2018-161. Washington: National Center for Education Statistics (2018). Available online at: https://nces.ed.gov/pubsearch/pubsinfo.asp?pubid=2018161 (Accessed June 29, 2021).

[26] Rantala A Pikkarainen M Miettunen J He H-G Pölkki T. The effectiveness of web-based mobile health interventions in paediatric outpatient surgery: a systematic review and meta-analysis of randomized controlled trials. J Adv Nurs. (2020) 76(8):1949–60. 10.1111/jan.14381 32281673

[27] Nguyen N Leveille E Guadagno E Kalisya LM Poenaru D. Use of mobile health technologies for postoperative care in paediatric surgery: a systematic review. J Telemed Telecare. (2020) 28(5):1357633X20934682. 10.1177/1357633X20934682 32605411

[28] Rackley S Bostwick JM. The pediatric surgeon-patient relationship. Semin Pediatr Surg. (2013) 22(3):124–8. 10.1053/j.sempedsurg.2013.04.002 23870204

[29] Litchfield I Shukla D Greenfield S. Impact of COVID-19 on the digital divide: a rapid review. BMJ Open. (2021) 11(8):e053440. 10.1136/bmjopen-2021-053440 34642200 PMC8520586

[30] Kan K Heard-Garris N Bendelow A Morales L Lewis-Thames MW Davis MM Examining access to digital technology by race and ethnicity and child health Status among Chicago families. JAMA Netw Open. (2022) 5(8):e2228992. 10.1001/jamanetworkopen.2022.28992 36018593 PMC9419010

